# A comparison of Chikungunya virus infection, progression, and cytokine profiles in human PMA-differentiated U937 and murine RAW264.7 monocyte derived macrophages

**DOI:** 10.1371/journal.pone.0230328

**Published:** 2020-03-12

**Authors:** Israel Guerrero-Arguero, Taalin R. Høj, E. Shannon Tass, Bradford K. Berges, Richard A. Robison

**Affiliations:** 1 Department of Microbiology and Molecular Biology, College of Life Sciences, Brigham Young University, Provo, Utah, United States of America; 2 Department of Statistics, College of Physical and Mathematical Sciences, Brigham Young University, Provo, Utah, United States of America; CEA, FRANCE

## Abstract

Chikungunya virus (CHIKV) is a mosquito-borne alphavirus that causes rash, fever and severe polyarthritis that can last for years in humans. Murine models display inflammation and macrophage infiltration only in the adjacent tissues at the site of inoculation, showing no signs of systemic polyarthritis. Monocyte-derived macrophages are one cell type suspected to contribute to a systemic CHIKV infection. The purpose of this study was to analyze differences in CHIKV infection in two different cell lines, human U937 and murine RAW264.7 monocyte derived macrophages. PMA-differentiated U937 and RAW264.7 macrophages were infected with CHIKV, and infectious virus production was measured by plaque assay and by reverse transcriptase quantitative PCR at various time points. Secreted cytokines in the supernatants were measured using cytometric bead arrays. Cytokine mRNA levels were also measured to supplement expression data. Here we show that CHIKV replicates more efficiently in human macrophages compared to murine macrophages. In addition, infected human macrophages produced around 10-fold higher levels of infectious virus when compared to murine macrophages. Cytokine induction by CHIKV infection differed between human and murine macrophages; IL-1, IL-6, IFN-γ, and TNF were significantly upregulated in human macrophages. This evidence suggests that CHIKV replicates more efficiently and induces a much greater pro-inflammatory cytokine profile in human macrophages, when compared to murine macrophages. This may shed light on the critical role that macrophages play in the CHIKV inflammatory response.

## Introduction

Chikungunya virus (CHIKV) is an alphavirus in the *Togaviridae* family. It consists of an outer membrane, an icosahedral capsid, and a positive sense RNA genome which encodes four structural proteins (C, E1, E2, and E3) and four non-structural proteins (nsP1, nsP2, nsP3, and nsP4) [1–3],. CHIKV is a reemerging disease that has caused major outbreaks in Southeast Asia, Africa, and more recently, in southern Mexico and other South American countries [4–6]. This disease is transmitted by two widely disseminated mosquito vectors from the *Aedes* genus (*Aedes aegypti* and *Aedes albopictus*) [7–11]. Recent outbreaks like the one in La Reunion were associated with the atypical mosquito vector, *Aedes albopictus* [12–14]. The expansion of the CHIKV vector unequivocally boosted CHIKV dissemination, which included its rapid expansion in 2015 throughout South America, and as far north as southern Mexico [9,15–18]. The main clinical symptoms are sudden fever, myalgia, rash and debilitating polyarthralgia [12,19,20]. The incubation period for this virus is between 3 and 7 days, and asymptomatic CHIKV cases range from 3–28% [21,22].

CHIKV disease in humans is marked by two phases. The acute phase usually lasts for 7–12 days with a plasma viral load of 10^6^−10^9^ pfu/mL [4]. Higher levels of viremia are more likely to be detected in newborn and elderly CHIKV patients who usually require hospitalization. During the chronic phase of this disease, long term persistence of anti-CHIKV IgM antibodies has been reported for up to 24 months [12,19,23]. This could be an indication of persistent viral antigenic presence providing a continuous stimulation of the humoral response. This may very well be the driving factor that leads to the development of chronic arthralgia, which can last for years [12,23].

The tropism of CHIKV in humans includes several human cell types such as primary epithelial and endothelial cells, monocyte-derived macrophages, and fibroblasts [24,25]. Similar to what happens with other alphaviruses, CHIKV-infected cells rapidly undergo apoptosis. Results from several biopsy studies have shown that CHIKV has a tendency to target muscle cells, skin fibroblasts, and joint tissue [25,26]. Additionally, there are also indications of endothelial tissue infections of the liver, spleen and brain [27–30]. Finally, the entry mechanism for CHIKV is still unclear, but there are indications that viral production is higher in human cells due to the interaction of viral proteins and certain human intracellular proteins. Interestingly, these interactions with mouse protein orthologs are lacking [31–34].

The lack of an effective vaccine or anti-viral treatment for CHIKV has resulted in substantial morbidity and considerable economic losses during outbreaks. In recent years, there have been some research efforts towards developing an animal model to build a better understanding of CHIKV pathogenesis; however, these rely on immune-deficient mice which develop swelling restricted to the inoculated foot, accompanied by higher levels of virus replication at the site of inoculation and little replication at distal sites [25,35–39]. This contrasts with the systemic infection seen in humans and the accompanying widespread arthritis. The reasons why mice are not the ideal model to study CHIKV pathogenesis are poorly understood.

In this study, we observed that CHIKV infection and replication efficiencies in human and murine monocytes are significantly different *in vitro*. Additionally, we observed significant differences in pro-inflammatory cytokine production induced by CHIKV infection in human and murine macrophage cell lines. These results suggest that CHIKV replication in macrophage cell lines varies by host species. This study did not explore further which factors may be related to the higher rates of virus production in human macrophages, but previous research has shown that viral-host interactions are species-selective[33].

## Materials and methods

### Cell culture and virus propagation

U937 and RAW264.7 cell lines were propagated in RPMI 1640 (HyClone Cat. No. SH30027.01) media supplemented with 10% heat-inactivated fetal bovine serum (FBS) (HyClone Cat. No. SH3008703), 10,000 units of Penicillin/Streptomycin (HyClone Cat. No. SV30010), 2mM L-glutamine, and 10mM of HEPES Buffer (HyClone Cat. No. SH3023701). Baby Hamster Kidney (BHK) cells were propagated in DMEM (HyClone Cat. No. 11966025) media supplemented with 10% heat-inactivated FBS, and 10,000 units of Penicillin/Streptomycin. The cells were cultured in T-75 culture flasks (Greiner Bio-One Cellstar Cat. No. 658170) at 37°C in an incubator with 5% CO_2_. U937 monocytes were transferred to 6-well tissue culture plates and induced to become adherent macrophage cells (5 X 10^5^ cells/mL) by exposure to 5ng/ml of phorbol 12-mystrate 13-acetate (PMA) (Thermofisher Cat. No. P1585) and incubated in 3 mL of RPMI 1640 complete media at 37°C for 24 hrs. CHIKV-LR strain was kindly provided by Dr. Jonathan Miner, Washington University, St. Louis, MO, and was propagated in Vero cells and stored for further use at -80°C. U937 cells were acquired from ATCC, while RAW264.7 cells stocks were donated by Dr. Kim O’Neill, Department of Microbiology and Molecular Biology, Brigham Young University, Provo, UT. Both U937 and RAW264.7 cell line stocks have been authenticated at University of Utah DNA sequencing core and University of Arizona Genetics core facilities, respectively.

### Viral quantification by plaque assay

CHIKV-LR stocks and supernatant of infected cultures were titrated in BHK cells. Virus samples were diluted in serial 10-fold dilutions in DMEM + 2% FBS and inoculated in 6-well plates which contained ~90% confluent BHK cultures. Inoculated 6-well plates were incubated for 1 hour to allow virus infection and then a 1:1 mix of 2X MEM + 8% FBS and low-melt agarose was used to overlay. Cultures were incubated for 3 days, fixed with 10% formalin and stained with crystal violet for plaques. Titer was calculated as Log10 PFU/mL and determined by the following equation: PFU/mL = (plaque count/well) * dilution factor / (mL inoculum).

### Infection assays

RAW264.7 and PMA-differentiated U937 macrophages were transferred to 12-well tissue culture plates at a cell density of 5 X10^4^ cells/mL and cultured overnight in complete medium at 37°C in 5% CO_2_. Cultures were infected using CHIKV-LR virus at various multiplicities of infection and incubated in a 37°C incubator with 5% CO_2_ for 2 hrs. Infected media was removed and cells were washed 3 times with PBS and fresh media was added and then incubated at the previously described conditions. Supernatant and intracellular RNA samples were taken at 2, 4, 6, 8, 12, 24, 36, and 48 hours’ post-infection and stored for plaque assay, or in Trizol Reagent for RNA extraction.

### RNA extraction

Intracellular RNA was extracted at previously mentioned time points using Trizol reagent (Thermofisher) and following the manufacturer’s directions. Viral RNA in supernatant was extracted using QIAamp Viral RNA Extraction following the manufacturer’s directions.

### RT-qPCR quantification of viral RNA

Intracellular lysate and supernatant of CHIKV infected cells at MOI of 0.1 and 5 was quantified by RT-qPCR using Applied Biosystems Taqman Fast Virus 1-Step Master Mix (Cat. No. 4444432) using a specific probe and primers for the CHIKV E1 gene. Initial reverse transcription was set at 50°C for 5 minutes; reverse transcription inactivation and initial denaturing stage at 95°C for 20 s and 40 cycles of amplification at 95°C for 5 s and 60°C for 30 s. Final primer and probe concentrations were 400nM and 250nM, respectively. A positive control plasmid was assembled by reverse transcribing CHIKV RNA using Life Technologies SuperScript IV Reverse Transcriptase kit (Cat. No. 18090050) using random hexamers as primers following the manufacturer’s directions. Amplification of the E1 gene was performed using primers containing a HindIII endonuclease restriction site in the reverse primer and an XbaI restriction site in the forward primer. Insertion of the PCR product into the pUC18 vector was performed by double restriction digest on the vector and insertion via HindIII-HF (NEB R3104S) and XbaI (NEB R0145S) restriction enzymes. The resulting plasmid, designated pUCE1, was transformed into *E*. *coli* chemically competent cells. Insertion of the E1 target sequence was confirmed by Sanger sequencing. A Ct standard curve for pUCE1 was done using nine 10-fold dilutions and obtaining the linear regression of the CT values; intercept of obtained experimental samples was analyzed and normalized to CHIKV genome copies per mL. Probe and primer sequences used in this method are shown in S1 Table.

### Flow cytometry

Infected PMA-differentiated U937 and RAW264.7 cultures were exposed to CHIKV virus at an MOI = 1 and incubated for 2 hours at 37°C, 5% CO_2_ atmosphere at an MOI of 1. After 2 hours, the cultures were thoroughly washed with PBS three times and fresh media was added and then incubated until 8 hpi. Cells were then Fc blocked for 30 min on ice with 10% human serum or mouse serum and 1% BSA in PBS. The cultures were then stained with either an anti-murine mCD11b-APC (ThermoFisher) or an anti-human hCD14-APC (ThermoFisher), and an anti-Chikungunya E1 protein antibody [CHK166; Antibody Research Corporation] previously conjugated with an Abcam Texas Red Conjugation kit (Cat. No. Ab195225) following the manufacturer’s recommendations. Cells where fixed with 10% formalin for at least 1 hour before removing them from the BSL-3 suite. Quantification of infected cells was performed using an BD Accuri C6 cytometer and analyzed using FlowJo version 10.5.3.

### RT-qPCR quantification of cytokine expression

Total RNA from PMA-differentiated U937 and RAW264.7 macrophages was reverse transcribed using Life Technologies SuperScript IV Reverse Transcriptase kit (Cat. No. 18090050) using random hexamers as RT primers following the manufacturer’s directions. ThermoFisher Scientific’s SYBR Select Master Mix was used for quantitative PCR assays. Specific primers for GAPDH, TNF, IL-1, IL-6, IL-10, IFN-α, IFN-γ and MCP-1 were designed to target the corresponding human and murine genes. GAPDH expression was used to normalize target mRNA expression, and fold expression changes were obtained by comparing CHIKV infected and uninfected cells using the ΔΔCT method. Probes and primers used in this method have been included in S1 Table.

### RT-qPCR quantification of Mxra8 expression

Total RNA from PMA-differentiated U937, undifferentiated U937 and RAW264.7 macrophages was reverse transcribed and PCR amplified following the same method previously described using Applied Biosystems Taqman Fast Virus 1-Step Master Mix (Cat. No. 4444432). Specific primers and probes were designed to target the human Mxra8 and GAPDH genes. GAPDH expression was used to normalize target mRNA expression, and fold expression changes were obtained by comparing PMA-differentiated U937 vs undifferentiated U937 cells using the ΔΔCT method. Probe and primer sequences used in these experiments are listed in S1 Table.

### Cytometric bead array

Supernatant samples containing secreted cytokines from infected cultures were harvested at 24 hpi and stored at -80°C. Samples were fixed in 10% formalin for at least 1 hour before removing them from the BSL-3 suite. Cytokine standard serial dilutions were prepared on the same day and a linear regression was used to correlate the sample values. Quantification of secreted cytokines was done using BD Biosciences Cytometric Bead Arrays for human cytokines (Cat. No. 551811) detecting TNF, IL-1, IL-6, IL-8, IL-10, and IL-12; and for murine cytokines (Cat. No. 552364) detecting IFN-γ, IL-6, IL-10, IL-12, and TNF. Sample preparation was done following the manufacturer’s directions and data was acquired in a BD Accuri C6 cytometer.

### Safety protocols

All of the experimental work involving infectious CHIKV was performed in a Biosafety Level 3 environment and complying with all Brigham Young University Institutional Biosafety Committee requirements which were approved in protocol IBC-2018-0028.

### Statistical analyses

Comparisons between groups were calculated in R (version 3.4.3) and analyzed with Welch's two-sample t-test which accounts for unequal variances between groups. We corrected for multiple comparisons using the Holm-Sidak method. *P* values of ≤0.05 were considered to be statistically significant. Graphics were generated using GraphPad Prism 8.0.1 for Windows, GraphPad Software, San Diego, California USA. Statistical results are included in S2 Table.

### Conflicts of interest

The authors declare no conflicts of interest. This research did not receive any specific grant from funding agencies in the public, commercial, or not-for-profit sectors.

## Results

### Chikungunya virus replicates to higher titers human macrophages than murine macrophages

**Fig 1:**
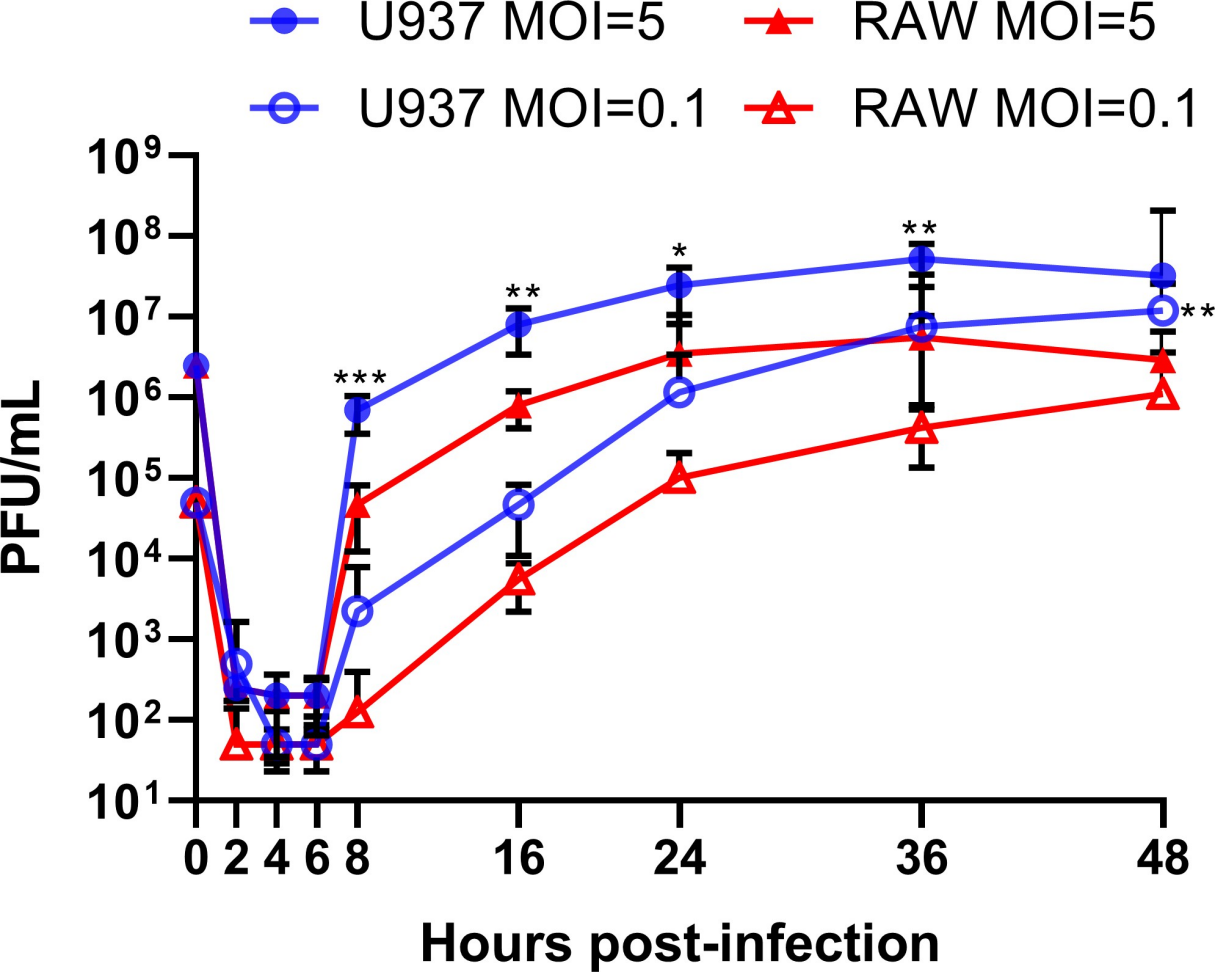
CHIKV replicates more efficiently in human macrophages than in murine macrophages. CHIKV infectious virus quantification was performed via plaque assay at the stated times (hpi). Data show mean values of three independent experiments with a total of n = 9, MOI = 0.1 and 5. Statistical significance was determined using multiple t-test corrected using Holm-Sidak method. *P<0.05; **P<0.01; ***P<0.001; NS, not significant.

**Fig 2:**
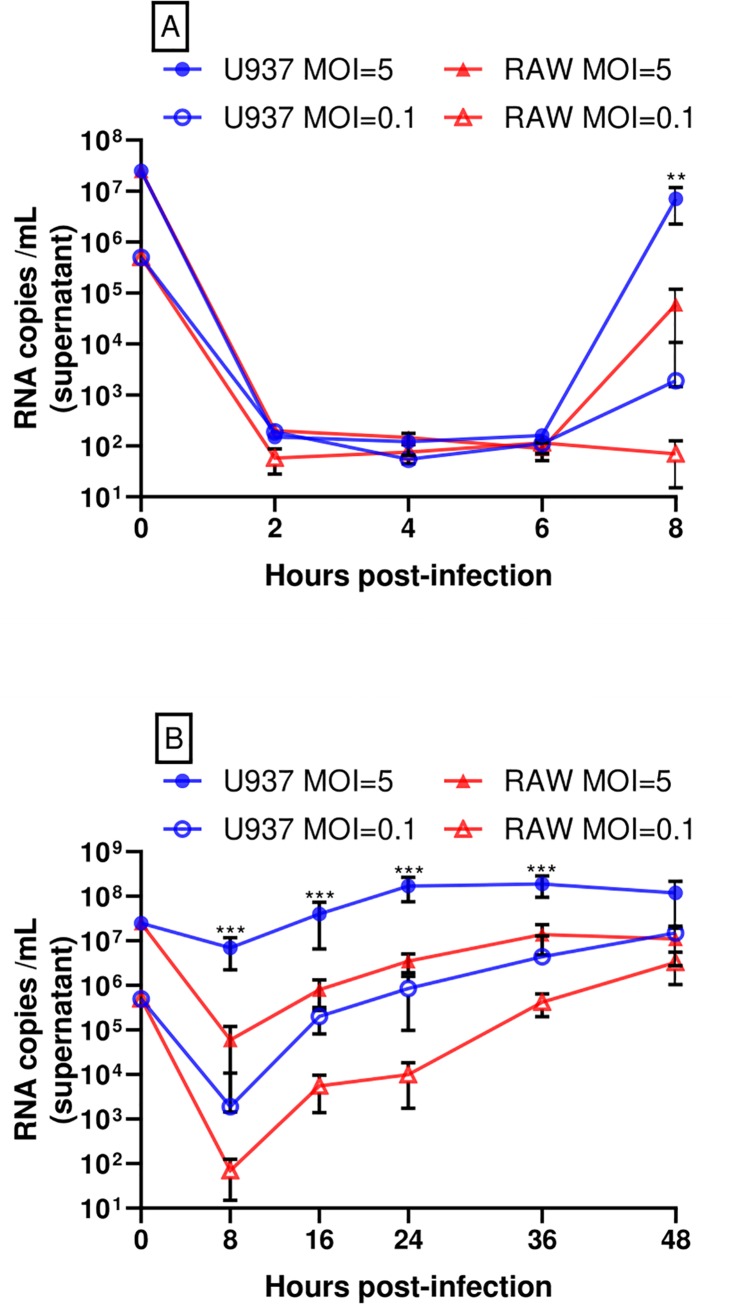
The CHIKV genome replicates more efficiently in human macrophages than in murine macrophages. A) RT-qPCR quantification of CHIKV RNA in supernatant samples collected from 2 to 8 hpi. B) RT-qPCR quantification of CHIKV RNA in supernatant samples collected from 8 to 48 hpi. Data show mean values of three independent experiments with a total of n = 9, MOI = 0.1 and 5. Statistical significance was determined using multiple t-test corrected using Holm-Sidak method. *P<0.05; **P<0.01; ***P<0.001; NS, not significant.

CHIKV has a wide range of tropism in human cells including fibroblasts, muscle cells and macrophages[40,41]. However, to our knowledge, there has not been a direct comparison of CHIKV replication efficiency and innate immune responses in human versus murine macrophages, which may shed light on the differences in CHIKV pathogenesis between these two species. The human PMA-differentiated U937 and murine RAW264.7 macrophages were infected with CHIKV-LR (La Reunion strain) at low and high multiplicity of infection (MOI), and viral supernatants were then titered by plaque assay. For both sets of infections, we observed an approximately 10-fold higher production of infectious CHIKV in PMA-differentiated U937 cells at 8, 16, 24, 36 and 48 hours post infection (hpi) when compared to RAW264.7 cells (Fig 1). Viral RNA quantification of supernatant samples confirmed our plaque assay findings. Viral RNA levels increased at 8 hpi with a 10-fold difference between human and murine cultures, regardless of initial MOI (Fig 2A). Replication of viral RNA and infectious virus at a high MOI in PMA-differentiated U937 cultures increases over time until it reaches a plateau at 24 hpi. This stationary phase is observed until 36 hpi in RAW 264.7 cultures. Viral replication (viral RNA and infectious virus) at a low MOI shows a constant increase of viral RNA and infectious virus until 48 hpi (Figs 1 and 2B).

**Fig 3:**
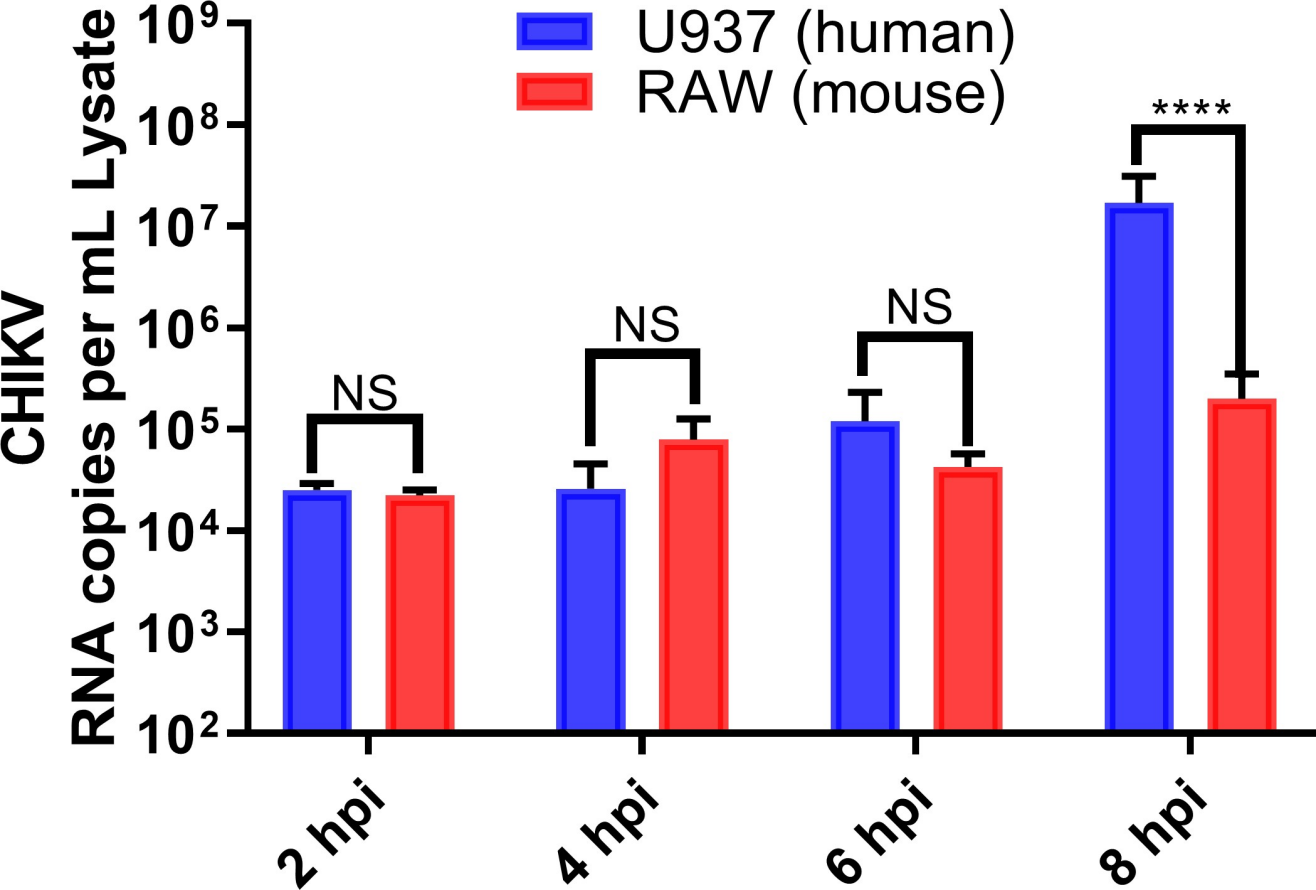
CHIKV genome levels in human and murine macrophages shortly after infection. Intracellular CHIKV RNA copies were quantified via RT-qPCR at stated times (hpi). Data show mean values of three independent experiments with a total of n = 9, MOI = 5. Statistically significant p values are denoted with an asterisk between compared groups. Statistical significance was determined using multiple t-test corrected using Holm-Sidak method. *P<0.05; **P<0.01; ***P<0.001; NS, not significant.

To determine if there is a difference in viral entry between murine and human macrophages, we measured both intracellular (Fig 3) and extracellular (Fig 2A) viral RNA during early time points of the first replication cycle. Our results showed that the majority of CHIKV RNA and infectious virus titer in supernatant decreases within 2 hpi in both cell lines, with no significant difference in intracellular viral RNA by cell type through 6 hpi (Fig 3). CHIKV infected PMA-differentiated U937 cells and RAW264.7 cells with similar efficiencies and it was not until 8 hpi that the amount of CHIKV RNA inside human macrophages increased about 2 logs greater than that in murine cells (Fig 3). These findings suggest a similar decrease in CHIKV titer in the supernatant but that it replicates better in human PMA-differentiated U937 macrophages.

**Fig 4:**
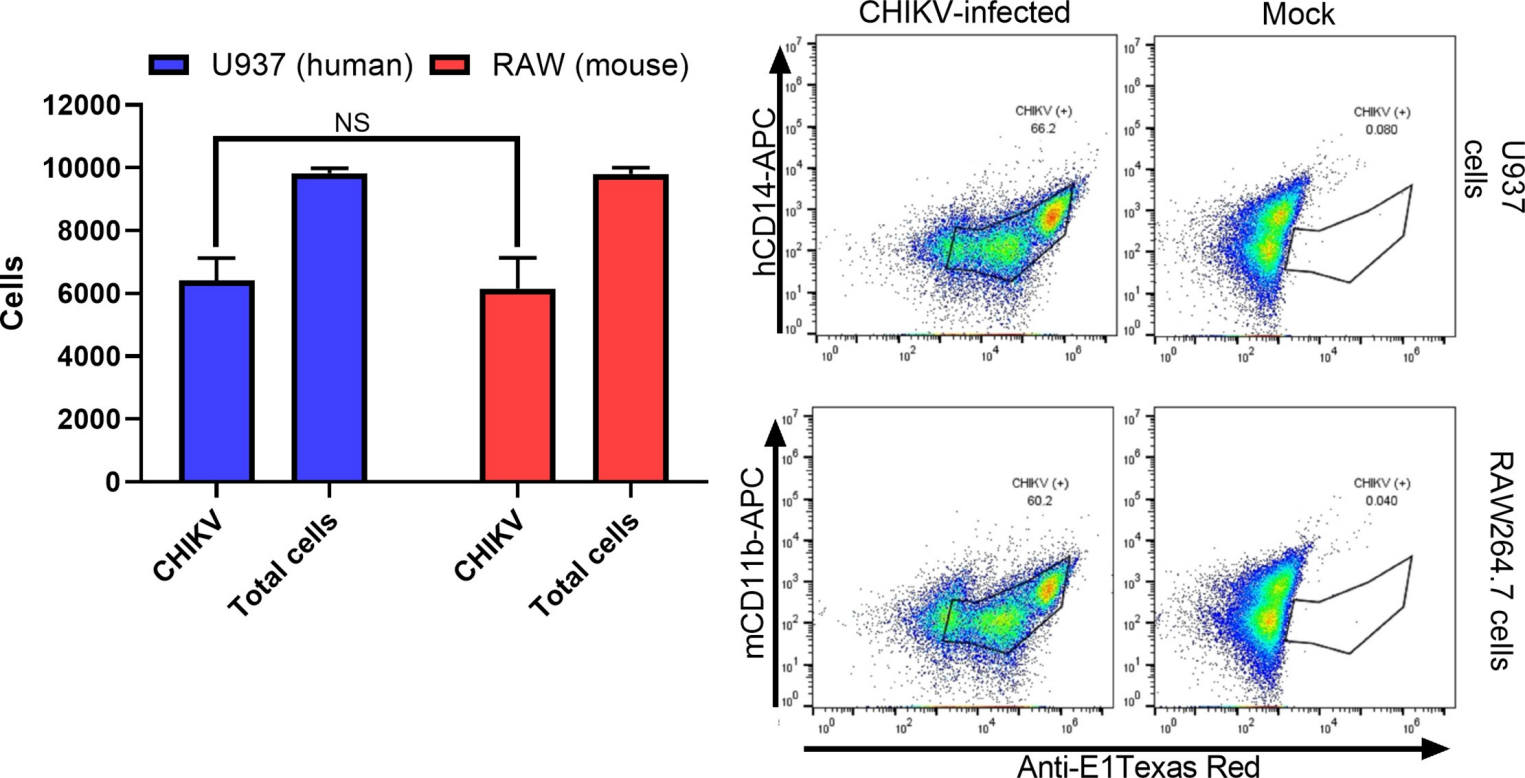
PMA-differentiated U937 and RAW264.7 cells display CHIKV envelope proteins at 6 hpi. PMA-differentiated U937 and RAW264.7 macrophages were exposed for 2 hours to CHIKV and then fixed and assayed at 8 hpi using flow cytometry and an anti-E1 protein fluorophore-conjugated monoclonal antibody. Data show mean values of three independent experiments with a total of n = 9, MOI = 1. Statistical significance was determined using multiple t-test corrected using Holm-Sidak method. *P<0.05; NS, not significant.

In addition, we explored the rate of productive CHIKV replication via flow cytometry. PMA-differentiated U937 and RAW264.7 cultures were infected at an MOI of 1 and quantified at 8 hpi using an anti-CHIKV antibody that targets the viral E1 glycoprotein. Results showed similar levels of E1 glycoprotein (an average of 60% positive cells) in both cell lines and no significant differences between human and murine macrophages (Fig 4).

These accumulated data suggest that virus production is higher in PMA-differentiated U937 human macrophages versus murine RAW264.7 macrophages, and that CHIKV titers decrease in the supernatant, regardless of the cell line.

### Production of pro-inflammatory cytokines following CHIKV infection shows species-specific differences

**Fig 5:**
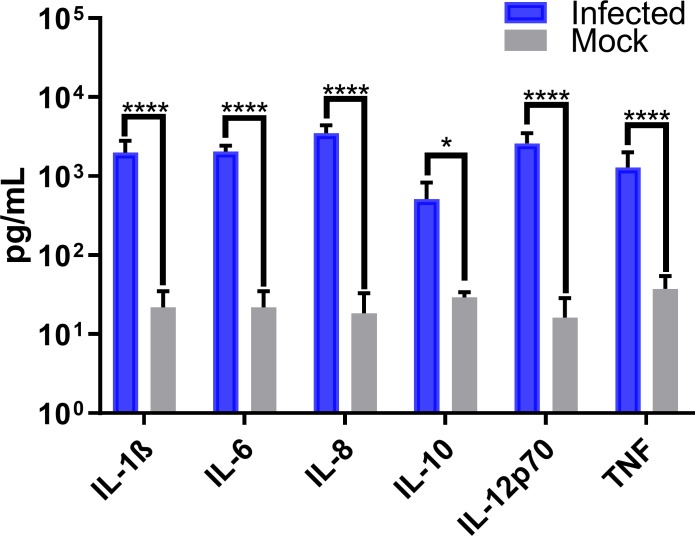
CHIKV infection induces pro-inflammatory cytokines in human macrophages. Secreted pro-inflammatory cytokine levels in CHIKV-infected PMA-differentiated U937 macrophages were quantified 24 hpi using cytometric bead arrays. Data show mean values of three independent experiments with a total of n = 9, MOI = 5. Statistical significance was determined using multiple t-test corrected using Holm-Sidak method. *P<0.05; **P<0.01; ***P<0.001; ****P<0.0001; NS, not significant.

Macrophages are one of the first lines of defense against infection and are responsible for the secretion of cytokine and chemokine signals to promote either anti- or pro-inflammatory pathways. Since systemic inflammation is a key difference in human versus murine infections, we examined a possible role in the mediation of this inflammation by cytokines secreted from infected human versus murine macrophages. CHIKV infection of PMA-differentiated U937 human macrophages showed a robust production of pro-inflammatory cytokines at 24 hpi when compared to PBS treatment as a mock-infection. Interleukins IL-1β, IL-6, IL-8, IL-10, IL-12p70, and Tumor Necrosis Factor (TNF) were significantly more abundant in CHIKV infected cell culture filtrates versus mock-infected ones (Fig 5).

**Fig 6:**
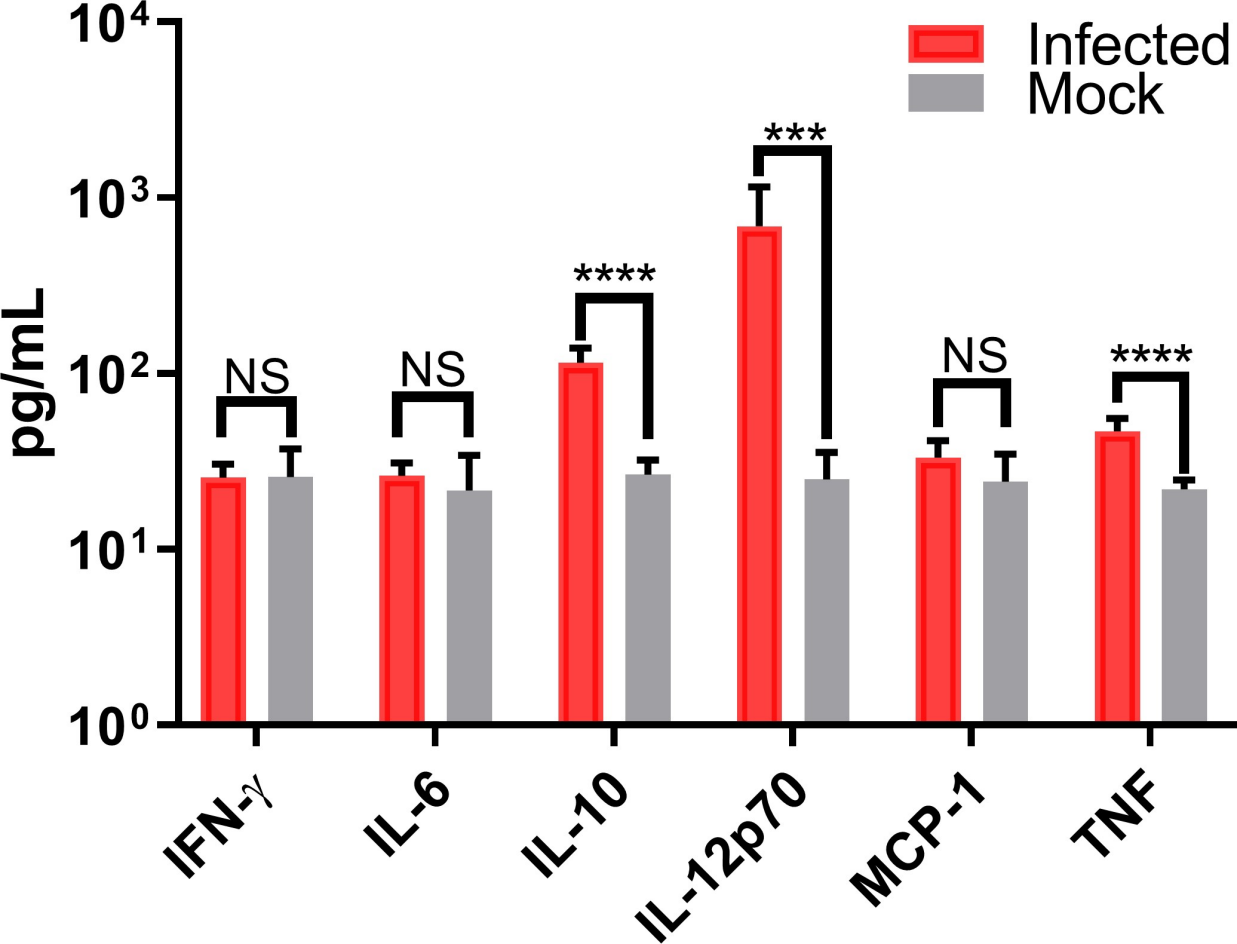
Cytokines induced in CHIKV-infected murine macrophages. Secreted cytokine levels in CHIKV-infected murine RAW264.7 macrophages were quantified 24 hpi using cytometric bead arrays. Data shows mean values of three independent experiments with a total of n = 9, MOI = 5. Statistical significance was determined using multiple t-test corrected using Holm-Sidak method. *P<0.05; **P<0.01; ***P<0.001; ****P<0.0001; NS, not significant.

**Fig 7:**
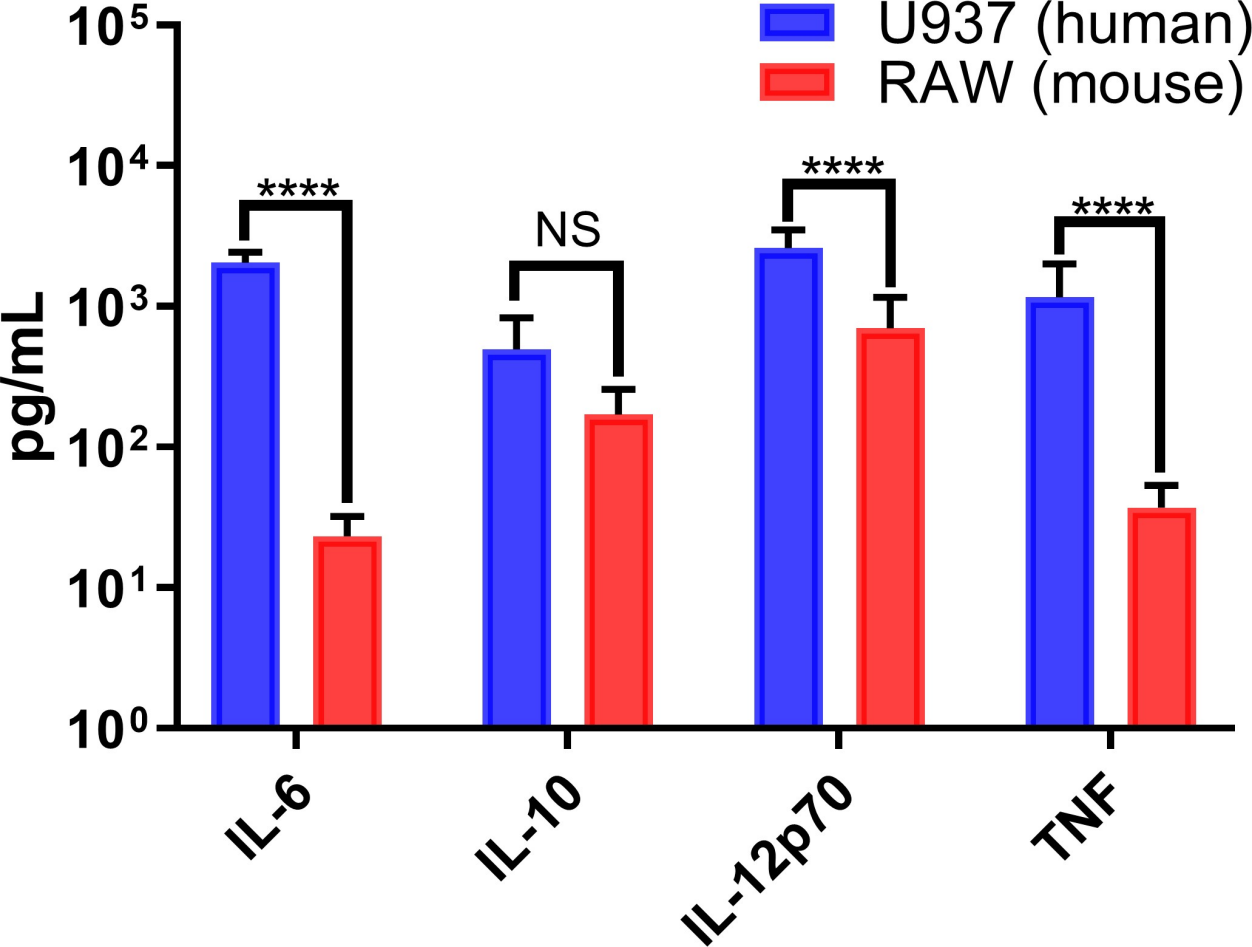
CHIKV-infected human macrophages show a more robust pro-inflammatory profile. Cytokine expression in CHIKV-infected human and murine macrophages was quantified by cytometric bead arrays at 24 hpi. Data show mean values of three independent experiments with a total of n = 9, MOI = 5. Statistical significance was determined using multiple t-test corrected using Holm-Sidak method. *P<0.05; **P<0.01; ***P<0.001; ****P<0.0001; NS, not significant.

Conversely, we observed that infection of murine macrophages showed a significant increased secretion of only two pro-inflammatory cytokines (IL-12p70, and TNF) and one anti-inflammatory cytokine (IL-10) in infected RAW264.7 cells, with similarly low levels of these cytokines in mock-infected cells (Fig 6). A direct comparison of secreted IL-6, IL-10, IL-12p70, and TNF concentrations in infected human and murine cultures indicate significant differences in all these cytokines but IL-10 (anti-inflammatory cytokine) (Fig 7). Cytokine responses in CHIKV-infected human patients have been extensively reported and can lead to a robust production of pro-inflammatory cytokines, compared to the relatively low levels observed in murine models[38,42–45].

**Fig 8:**
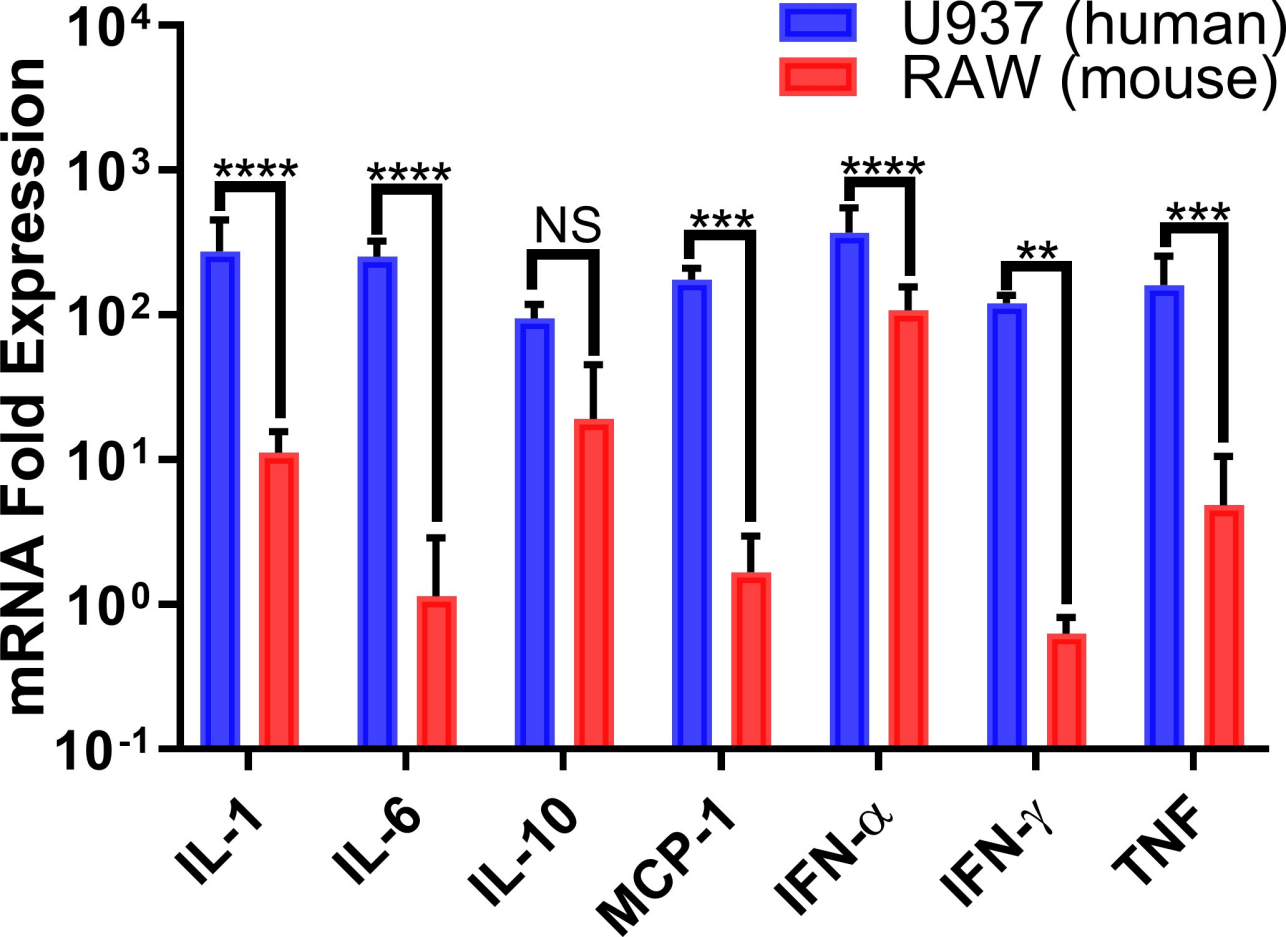
CHIKV infection upregulates pro-inflammatory cytokines mainly in human macrophages. Cytokine mRNA expression levels in CHIKV-infected human and murine macrophages was quantified by RT-qPCR at 24 hpi. Results were normalized relative to GAPDH expression levels. Data shows mean values of three independent experiments with a total of n = 9, MOI = 5. Statistical significance was determined using multiple t-test corrected using Holm-Sidak method. *P<0.05; **P<0.01; ***P<0.001; ****P<0.0001; NS, not significant.

We also explored the differences in pro-inflammatory cytokine induction between CHIKV-infected human and murine macrophages by comparing the RT-qPCR (relative quantification) values for relevant pro-inflammatory cytokine mRNAs in human and murine cells, which confirmed differences in the expression of pro-inflammatory cytokines as measured by bead arrays. CHIKV-infected PMA-differentiated U937 and RAW264.7 macrophages displayed different gene expression profiles during CHIKV infection peak activity (24 hpi). mRNA levels for IL-1, IL-6, IFN-α, IFN-γ, MCP-1, and TNF were significantly higher in human cells when compared to the expression levels of their murine counterparts (Fig 8). Again, IL-10 mRNA levels were similar in both human and murine cells, confirming our previous findings.

### Mxra8 alphavirus entry mediator is upregulated in PMA-differentiated U937 macrophages

**Fig 9:**
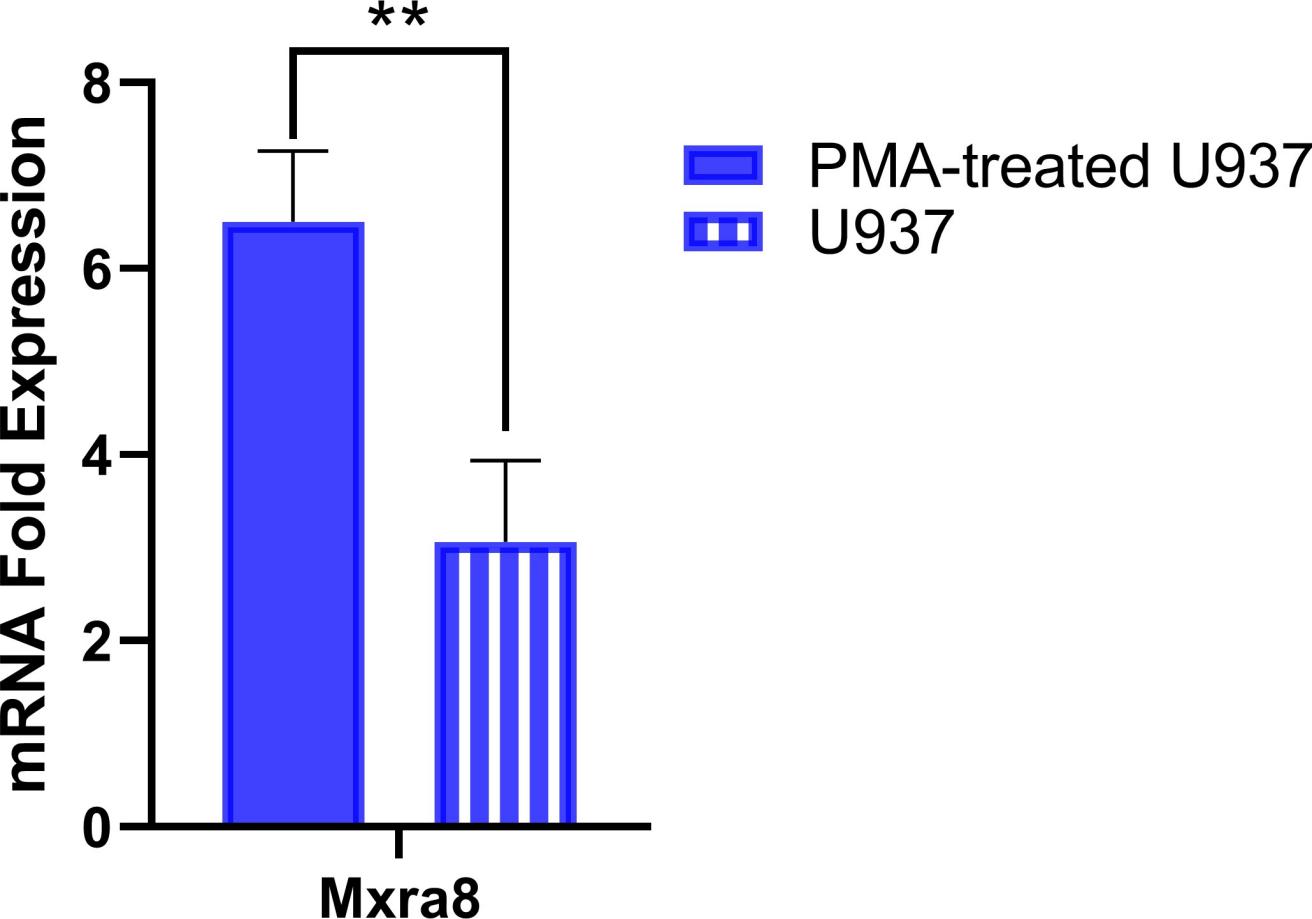
Mxra8 expression levels are higher in PMA-differentiated U937 macrophages. Mxra8 mRNA expression levels in PMA-differentiated macrophages and undifferentiated U937 monocytes was quantified by RT-qPCR. Results were normalized relative to GAPDH expression levels. Data shows mean values of three independent experiments with a total of n = 9. Statistical significance was determined using a Welch two-sample t-test. **P<0.01.

The matrix remodeling associated 8 (Mxra8) protein has been recently identified as an entry mediator for multiple arthritogenic alphaviruses, including CHIKV[46,47]. Therefore, we assayed the expression levels of Mxra8 in PMA-differentiated U937 macrophages and undifferentiated U937 monocytes using RT-qPCR (Fig 9). PMA-differentiated U937 cells showed a significant expression increase over undifferentiated U937 cells (P = 0.0097).

## Discussion

CHIKV has been shown to infect a wide variety of different cell types including immune, epithelial and endothelial cells. Murine *in vivo* and *in vitro* infection studies have shown that CHIKV infects brain tissue and glial cells [48], dendritic cells, macrophages [49], and epithelial cells[2]. In humans, CHIKV infects endothelial, epithelial, fibroblast, muscle satellite and macrophage cells [21,32,35,39,50,51]. Despite the similarities in cell types targeted across species, the stark differences in immune responses to infection between human and murine models are significant obstacles in using murine models to aid in understanding CHIKV pathogenesis [52].

CHIKV infection has been studied extensively in many murine models, however, these models have several inconsistencies when compared to symptoms present in human infections. Common manifestations seen in infected human patients like persistent polyarthritis, and chronic inflammation are not observed in current murine models [38,39,53]

The mechanisms involved in the dissemination of CHIKV within the host remain largely unknown. Macrophages seem to be involved in joint inflammation [43] in humans and non-human primate models, since significant infiltration of these cells has been detected in joints during the acute phase, and long after virus clearance from the blood [27]. To our knowledge, there has not been a study that directly compares CHIKV replication in human and murine monocytes or activated macrophages, and the differences in cytokine responses induced in these cells following CHIKV infection.

CHIKV infection of murine RAW264.7 has been previously explored[54]. This report compared viral infectivity and cytokine induction between RAW264.7 and a CTLL astrocyte cell line. CHIKV only infected 5% of the RAW264.7 populations whereas 100% of the CTLL cells were successfully infected at an MOI of 1. Additionally, viral kinetics in this mentioned study showed that CHIKV RNA replication produced higher titers in CTLL cells compared to RAW264.7. Cytokine response showed upregulation of pro-inflammatory markers like TNF-α, IFN- α and ISG-56 at 24 hpi.

Using both RT-qPCR and plaque assays, we observed that about 10-fold higher levels of CHIKV was produced in PMA-differentiated U937 macrophages when compared to those produced in infected RAW264.7 macrophages (Figs 1, 2A and 2B). Our CHIKV replication curves in both PMA-differentiated U937 and RAW264.7 macrophages correlated with the results reported by others, where CHIKV virions and viral RNA increased steadily, reaching a peak at 24 hpi, and then decreasing slightly until the end of the experiment at 48 hpi [40].

In comparison, our study showed poor innate immune response in cytokine gene expression and secretion, delayed virus production and lower titers, regardless of MOI. Our plaque assay, RT-qPCR, and flow cytometry results suggest that CHIKV infects and replicates in both human and murine macrophage cell lines within the first 8 hpi. However, CHIKV titer at 8 hpi is significantly lower in RAW264.7 versus PMA-differentiated U937 macrophages (Figs 1 and 2A). Additionally, delayed production of infectious virus titer and viral mRNA in supernatant was displayed in RAW264.7 macrophages from 8hpi until 48 hpi (Figs 1 and 2B). These results were consistent both at a MOI = 5 and MOI = 0.1. We decided to explore CHIKV RNA replication efficiencies within the first replication cycle to better understand these species-related differences. We quantified the RNA viral titer from our inoculum and tracked its presence in the cell supernatant. Within the first 6 hpi, we observed no significant differences in viral RNA levels between species, inside the infected macrophages (Fig 3). Flow cytometry quantification of CHIKV infected cells at 8 hpi showed similar levels of E1 glycoprotein on the cell membranes of both human and murine cells (Fig 4), indicating that about 60% of both human and murine macrophage cell lines were infected. Our intention was to quantify the amount of CHIKV infected cells at a MOI = 1 and assess a ratio of positive infected cells close to the first viral outpouring. As previously mentioned, CHIKV production seems to be tied to the species of the host cell.

Judith, et al explored CHIKV viral production in HeLa and MEF cells. Their results indicated that human NDP52, but not the murine orthologue, interacts with CHIKV nsP2, and that inhibiting synthesis of this protein reduces viral production [33]. An additional study performed in yeast indicated that nsP2 interacts with heterogeneous nuclear ribonucleoprotein K (hnRNP-K) and ubiquilin 4 (UBQLN4), resulting in CHIKV replication in vitro [2]. In total, this study identified 30 interactions between nsP2, nsP4 and E3 viral proteins and various human host factors. However, they also acknowledged that no cellular partners were found for the rest of the CHIKV proteins, which may reflect the technical limitations of their yeast two-hybrid system.

It is also noteworthy to mention the role of viral proteins with intracellular host factors, like NDP52, wherein murine cells CHIKV protein synthesis is inhibited, whereas in humans, virus production is enhanced[2,33]. It becomes clearer that many factors between these cell lines are responsible for delayed or enhanced virus replication, which appear to be unique to the host species. It is possible that the presence of Mxra8 enhances CHIKV binding and fusion to the host cell and once in the cytoplasm species-specific interactions between viral proteins and host cell machinery further influence viral replication in the host cell.

We proceeded to explore the cytokine profiles of infected human and murine macrophages to better understand inflammation differences between these species. CHIKV infection in human macrophages triggered secretion of the pro-inflammatory cytokines IL-1β, IL-6, IL-8, IL-12p70, and TNF (Fig 5). These data are consistent with pro-inflammatory cytokine profiles of infected patients and non-human primate models [27,52,55].

Interestingly, in Kumar et al increased levels of TNF-α in CHIKV infected RAW264.7 macrophages decreased apoptosis susceptibility[56]. Additionally, they observed that CHIKV infected RAW264.7 macrophages did not produce significant levels of several interleukins, including IL-10. This lead to the conclusion that CHIKV infection in RAW264.7 macrophages leads to poor innate immune response, high TNF- α expression, and low apoptotic activity.

However, we observed a different cytokine response in murine macrophages, with only IL-10, IL-12p70 and TNF showing significant differences from uninfected controls (Fig 6). The absence of IFN-γ indicates a lack of monocyte/macrophage activation but the presence of high levels of IL-12p70 indicate that the exposed macrophages have recognized the presence of a pathogen. These results may indicate that RAW264.7 cells require interaction with IL-12-activated T_H_1 cells, which were not present in our *in vitro* assays. In contrast, IFN-γ mRNA levels indicate upregulation in human PMA-differentiated U937 macrophages suggesting macrophage activation. Additionally, IL-12p70 levels in PMA-differentiated U937 macrophages indicate a possible autocrine induction of IFN-γ upregulation[57,58].

While we did not measure human MCP-1 by bead array, we did measure its mRNA levels by RT-qPCR. This cytokine plays an important role in macrophage recruitment and it was expressed at higher levels in CHIKV-infected PMA-differentiated U937 human macrophages, compared to the murine cell line (Fig 8). This could lead to fewer infections of circulating monocytes, effectively stalling the systemic spread of CHIKV in mice.

Macrophage infiltration of affected tissues has been extensively reported in CHIKV and other arthritis-causing alphaviruses [21,59–61]. Mice with macrophage recruitment deficiencies showed significant reductions of tissue infiltration and inflammation during CHIKV infection[62]. Other studies also confirmed that the inhibition of MCP-1 reduced inflammatory responses and infiltration of macrophages in CHIKV-infected mice[43,63]. The lack of expression of this important macrophage chemokine attractant by murine RAW264.7 cells could also contribute to the inability of murine animal models to mimic the polyarthritis which is a hallmark of many CHIKV human infections. An increase in TNF secretion by infected human macrophages indicates a robust systemic inflammatory response, whereas in contrast, murine macrophages display a mild induction of TNF production (Figs 6 and 7). The induction of pro-inflammatory cytokines in PMA-differentiated U937 human macrophages was significantly higher than that of murine RAW264.7 macrophages (Fig 7). Pro-inflammatory cytokine mRNA expression levels in CHIKV-infected human and murine macrophages showed similar species-specific differences. Upregulation of IFN-α in human and murine macrophages indicated that the cells recognized a viral infection and initiated antiviral signaling (Fig 8). However, murine macrophages did not significantly upregulate the expression of IL-6 or IFN-γ, which are critical factors for systemic inflammation. mRNA levels in murine RAW264.7 macrophages showed increases in the expression of MCP-1 (~2-fold), IL-1(~10-fold), IFN-α (over 100-fold), IL-10 (over 10-fold), and TNF (~5-fold), which indicated a discrete upregulation that correlated with our secreted cytokine results.

Interestingly, we observed significant gene expression upregulation and cytokine secretion of IL-10 in CHIKV infected RAW264.7 macrophages compared to mock infected (P<0.00001 and P<0.001, respectively) (Figs 6 and 8). However, gene expression and secretion of IL-10 in CHIKV infected RAW264.7 macrophages showed no significant differences versus PMA-differentiated U937 macrophages (Figs 7 and 8).

In PMA-differentiated U937 macrophages displayed upregulation in all the screened cytokines including: IL-1 (~150 fold), IL-6 (~150 fold), MCP-1 (~100 fold), IFN-α (~150 fold), IFN-γ (~100 fold), and TNF (~100 fold), indicating a more robust activation of pro-inflammatory cytokine response during CHIKV infection (Fig 8). The induction of these Th1 pro-inflammatory cytokines in PMA-differentiated U937 macrophages, is similar to a previous report that observed Th1, Th2 and Th17 cytokine profile induction of undifferentiated U937 cells during CHIKV and Mayaro virus infection[64].

The role of Mxra8 as an arthritogenic alphavirus receptor was recently reported[46,47]. Deletion of this gene or blocking of the surface protein in human and murine cells resulted in reduced levels of viral infection. It was shown that Mxra8 binds directly to CHIKV E2 protein and enhances virus attachment and internalization into the cells. The increased presence of Mxra8 mRNA in PMA-differentiated U937 macrophages could explain why these cells are significantly more permissive to CHIKV infection (Fig 9) [65,66]. Other publications have shown that CD14+ peripheral blood mononuclear cells (PBMC) are susceptible to CHIKV infection, however, this report seems to encompass all CD14+ mononuclear cells[45], whereas the most relevant mononuclear subset to CHIKV infection is differentiated macrophages[27,62,67]. It has been suspected that CHIKV infection in humans induces a pro-inflammatory cytokine profile (Th1 and Th17) which in turn triggers persistent joint pain and polyarthritis pathology not only by activating host inflammatory cytokines, but also by the virus itself hijacking resident tissue macrophages, as has previously been described for other alphaviruses, such as Ross River Virus and Mayaro virus[24,59,61,62,68]. Here, we examined whether the outcomes of CHIKV infection of macrophage lines from different species would differ, and if so, whether these differences could help explain the failure of the murine model to mimic polyarthritis and chronic inflammation seen in humans. We can conclude that CHIKV infects macrophages from both species, but replicates more efficiently in human macrophages.

**Fig 10:**
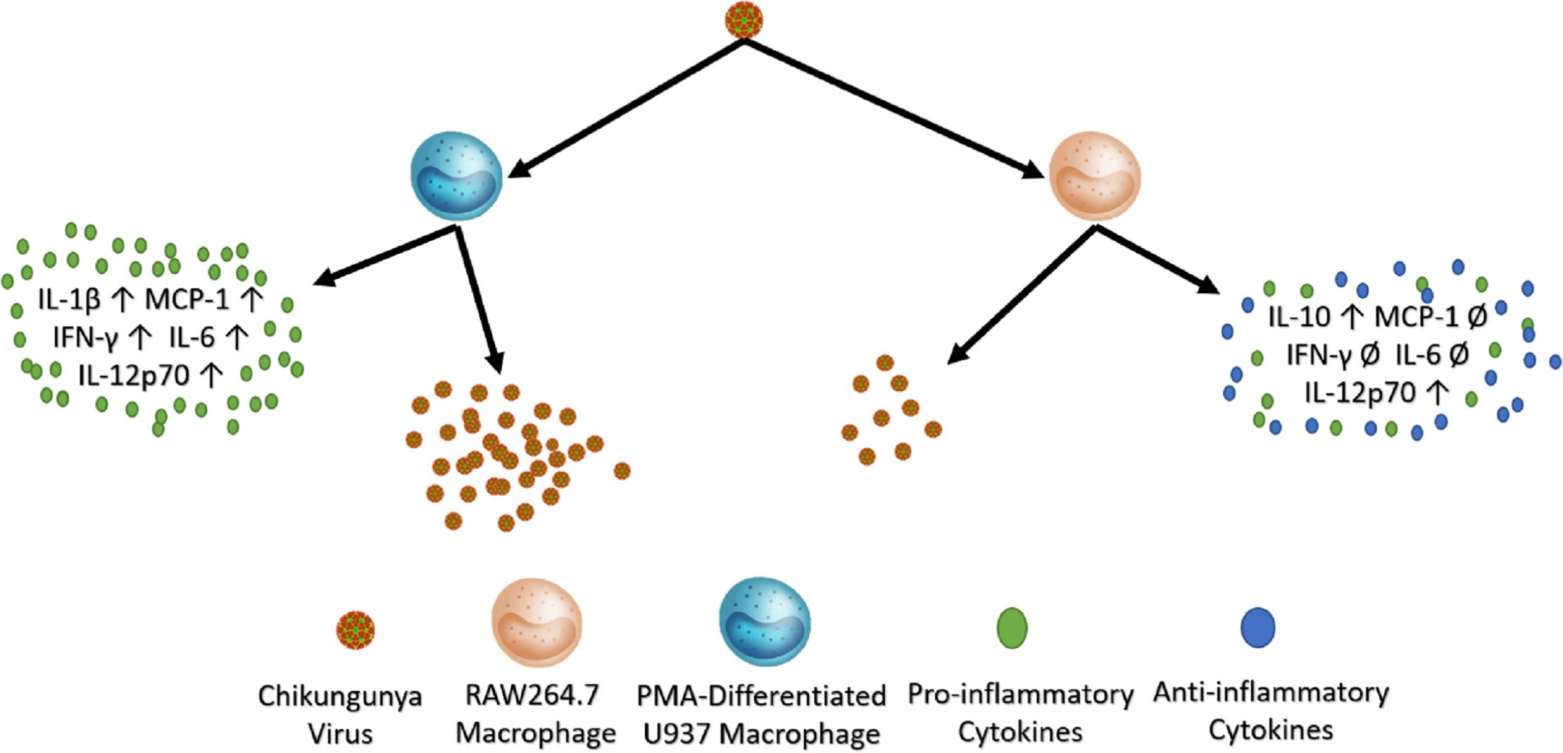
A graphic summary of CHIKV infection in human PMA-differentiated U937 macrophages and murine RAW264.7 macrophages. CHIKV infection in PMA- differentiated U937 macrophages produces higher amounts of virions and induces a more vigorous pro-inflammatory cytokine response. CHIKV infection in RAW264.7 cells results in lower quantities of virions and induction of more anti-inflammatory cytokines.

Also, the cytokine profile of infected murine macrophages indicates the beginnings of an immune response towards infection by triggering the expression and secretion of IFN-α and IL-12p70. However, this stands in contrast to the robust pro-inflammatory cytokine response that infected human macrophages display. A graphical representation of these results has been summarized in Fig 10. Further research is needed to identify which intracellular interactions between host factors and viral components are most important for viral replication in human cells. Finally, our results suggest that the addition of human macrophages to a murine model, such as is available in humanized mouse models, could potentially bring the necessary components together to recapitulate the chronic polyarthritis seen in human infections.

## Supporting information

S1 Table Primer and probe sequences. (PDF)

S2 Table Statistical analysis of the results. (PDF)
