# Supplementary material for: A comparison of Chikungunya virus infection, progression, and cytokine profiles in human PMA-differentiated U937 and murine RAW264.7 monocyte derived macrophages
Source: PLoS One. 2020 Mar 12;15(3):e0230328. doi: 10.1371/journal.pone.0230328 (PMC7067478; doi:10.1371/journal.pone.0230328)
Supplement: S1 Table — (PDF) [file pone.0230328.s001.pdf]

S1 Table. Primer and Probe Sequences

	Human	*Murine*
Name	5'->3' Nucleotide Sequence	5'->3' Nucleotide Sequence
IL-1 For	ATGTCTCTGCAAAAGACCCCT	CAACCAACAAGTGATATTCTCCATG
IL-1 Rev	GGTAGCAGTTTGGGCTTGTGT	GATCCACACTCTCCAGCTGCA
IL-6 For	ACTCACCTCTTCAGAACGAATTG	CTGCAAGAGACTTCCATCCAG
IL-6 Rev	CTGCAAGAGACTTCCATCCAG	AGTGGTATAGACAGGTCTGTTGG
IL-10 For	TACCACCTCCCGAAAATGTCA	GCTGGACAACATACTGCTAACC
IL-10 Rev	CCCAGTCTGAATGCTCATCTG	ATTTCCGATAAGGCTTGGCAA
IFN-α For	CACTTCTAGACAAATTCTACACTG	TGAAGGACAGGAAGGACTTTG
IFN-α Rev	GGATAGAGTCCACATTCATCAG	GAATGAGTCTAGGAGGGTTGT
IFN-γ For	CCAACGCAAAGCAATACATGA	TCAAGTGGCATAGATGTGGAAGAA
IFN-γ Rev	CCTTTTTCGCTTCCCTGTTTTA	GCTGGACAACATACTGCTAACC
TNF-α For	GGAGAAGGGTGACCGACTCA	AATTCGAGTGACAAGCCTGTAGC
TNF-α Rev	CTGCCCAGACTCGGCAA	AGTAGACAAGGTACAACCCATCG
MCP-1 For	CAGCCAGATGCAATCAATGCC	TAAAAACCTGGATCGGAACCAAA
MCP-1 Rev	TGGAATCCTGAACCCACTTCT	GCATTAGCTTCAGATTTACGGGT

	Other	
	5'->3' Nucleotide Sequence	
E1 Cloning F	GCAGCAAAGCTTAGAACAGCTAAAGCGGCCAC	Cloning primers for CHIKV's E1 gene
E1 Cloning R	GCAGCATCTAGACCTGCTGAACGACACGCATAG	
Mxra8-For	TACACCTGCAACCTGCACC	
Mxra8-Rev	ACCTGTTGAGCCTCCTCCAC	
Mxra8-Pro	FAM-ACTACTGCCACCTCTACGAGAGCCTGGCCGTCCG-BHQ1	
GapDH-Probe	Cy3-ATCGGTCGCCTGGTCCTGCGCGCCTGCATGGA-BHQ2	Designed to amplify human and murine sequences
GapDH-For	TGTGGGCATCAATGGATTTGG	
GapDH-Rev	ACACCATGTATTCCGGGTCAAT	
E1 For	TCGACGCGCCCTCTTTAA	CHIKV E1 gene
E1 Rev	ATCGAATGCACCGCACACT	
E1 Probe	FAM-ACCAGCCTGCACCCATTCCTCAGAC-TAM	
