# Supplementary material for: A comparison of Chikungunya virus infection, progression, and cytokine profiles in human PMA-differentiated U937 and murine RAW264.7 monocyte derived macrophages
Source: PLoS One. 2020 Mar 12;15(3):e0230328. doi: 10.1371/journal.pone.0230328 (PMC7067478; doi:10.1371/journal.pone.0230328)
Supplement: S2 Table — (PDF) [file pone.0230328.s002.pdf]

S2 Table. Statistical Analysis of the Results

Figure 1

Hpi	Mean PMA-U937 MOI=5	Mean RAW264.7 MOI=5	SE of Difference	P-value	Adjusted P-value
0	2500000	2500000	60310	>0.999999	>0.999999
2	250	250	31.62	>0.999999	>0.999999
4	200	200	60.01	>0.999999	>0.999999
6	200	200	58.9	>0.999999	>0.999999
8	700000	47000	116062	0.000038	0.000341
16	8000000	800000	1547869	0.000266	0.001861
24	24500000	3500000	5955456	0.002804	0.016707
36	52000000	5500000	9676521	0.000194	0.001552
48	32200000	2900000	58582423	0.623778	0.992463
	Mean U937 MOI=0.1	Mean RAW264.7 MOI=0.1	SE of Difference	P-value	Adjusted P-value
0	50000	50000	3255428	>0.999999	>0.999999
2	500	50	3255428	0.99989	>0.999999
4	50	50	3255428	>0.999999	>0.999999
6	50	50	3255428	>0.999999	>0.999999
8	2230	125	3255428	0.999485	>0.999999
16	47000	5500	3255428	0.989847	>0.999999
24	1150000	101200	3255428	0.747791	0.999935
36	7500000	420000	3255428	0.031276	0.224464
48	12000000	1100000	3255428	0.001038	0.009303

Figure 2A

Hpi	Mean PMA-U937 MOI=5	Mean RAW264.7 MOI=5	SE of Difference	P-value	Adjusted P-value
0	25000000	25000000	2126766	>0.999999	>0.999999
2	150	199	2126766	0.999982	>0.999999
4	120	145	2126766	0.999991	>0.999999
6	160	89	2126766	0.999973	>0.999999
8	7000000	60000	2126766	0.001621	0.008081
	Mean U937 MOI=0.1	Mean RAW264.7 MOI=0.1	SE of Difference	P-value	Adjusted P-value
0	500000	500000	4795	>0.999999	>0.999999
2	188	58	48.41	0.01626	0.078697
4	55	75	30.41	0.520147	0.946981
6	111	114	59.46	0.960388	0.998431
8	1900	70	8800	0.837892	0.99574

Figure 2B

Hpi	Mean PMA-U937 MOI=5	Mean RAW264.7 MOI=5	SE of Difference	P-value	Adjusted P-value
0	25000000	25000000	69633247	>0.999999	>0.999999
8	7000000	60000	69633247	0.920818	0.99373
12	40000000	800000	69633247	0.574782	0.923116
24	170000000	3500000	69633247	0.018748	0.090291
36	190000000	14000000	69633247	0.01312	0.076182
48	120000000	11000000	69633247	0.120793	0.402463
	Mean U937 MOI=0.1	Mean RAW264.7 MOI=0.1	SE of Difference	P-value	Adjusted P-value
0	500000	500000	3685022	>0.999999	>0.999999
8	1900	70	3685022	0.999605	>0.999999
12	200000	5500	3685022	0.958016	0.999926

24	850000	10000	3685022	0.82017	0.998954
36	4400000	420000	3685022	0.282827	0.810277
48	15000000	3300000	3685022	0.002014	0.012025

Figure 3

Hpi	Mean PMA-U937 MOI=5	Mean RAW264.7 MOI=5	SE of Difference	P-value	Adjusted P-value
2	25125	22229	2333279	0.999013	0.999981
4	25889	78433	2333279	0.982104	0.999981
6	120150	42362	2333279	0.973508	0.999981
8	16939492	199301	2333279	<0.000001	<0.000001

Figure 4

	Mean PMA-U937 MOI=1	Mean RAW264.7 MOI=1	SE of Difference	P-value	Adjusted P-value
Total cells	9825	9802	85.87	0.791254	0.791254

Figure 5

Cytokine	Mean CHIKV Infected	Mean Mock Treated	SE of Difference	P-value	Adjusted P-value
IL-1ß	1980	21.78	237.2	<0.000001	<0.000001
IL-6	2045	21.89	237.2	<0.000001	<0.000001
IL-8	3509	18.44	237.2	<0.000001	<0.000001
IL-10	514.8	29.11	237.2	0.043321	0.043321
IL-12p70	2596	16.22	237.2	<0.000001	<0.000001
TNF	1288	37.33	237.2	<0.000001	0.000002

Figure 6

Cytokine	Mean CHIKV Infected	Mean Mock Treated	SE of Difference	P-value	Adjusted P-value
IFN-γ	25.67	25.78	4.143	0.978933	0.978933
IL-6	26.11	21.56	4.517	0.328219	0.54871
IL-10	115.4	26.67	8.24	<0.000001	<0.000001

IL-12p70	688.3	25.11	153.4	0.000523	0.002092
MCP-1	33.33	24.22	4.412	0.055515	0.157469
TNF	47	21.89	3.089	<0.000001	0.000002

Figure 7

Cytokine	Mean PMA-U937	Mean RAW264.7	SE of Difference	P-value	Adjusted P-value
IL-6	2045	23.22	235.7	<0.000001	<0.000001
IL-10	492.5	171.1	235.7	0.177368	0.177368
IL-12p70	2596	694.6	235.7	<0.000001	<0.000001
TNF	1165	37	235.7	0.00001	0.000021

Figure 8

Cytokine	Mean PMA-U937	Mean RAW264.7	SE of Difference	P-value	Adjusted P-value
IL-1	275.8	11.18	42.83	<0.000001	<0.000001
IL-6	251.2	1.14	42.83	<0.000001	<0.000001
IL-10	95.27	19.19	42.83	0.08014	0.08014
MCP-1	175.5	1.677	42.83	0.00013	0.00052
IFN-α	371.1	107.1	44.92	<0.000001	<0.000001
IFN-γ	120.6	0.6283	42.83	0.006641	0.013239
TNF	161.3	4.833	44.92	0.000868	0.002603

Figure 9

	Mean PMA-U937	Mean U937	SE of Difference	P-value	
PMA-treated U937 vs. U937	6.506	3.061	1.157	0.0318	0.0097
